# Performance of the new SLICC classification criteria in juvenile systemic lupus erythematosus, in two observation periods: a Brazilian study

**DOI:** 10.1186/1546-0096-12-S1-P318

**Published:** 2014-09-17

**Authors:** Adriana R Fonseca, Maria Ignez C Gaspar-Elsas, Marcelo Land, Sheila K Oliveira

**Affiliations:** 1 Pediatric Rheumatology, Universidade Federal do Rio de Janeiro, Brazil; 2 Immunology, Fundação Oswaldo Cruz/Instituto Fernandes Figueira, Brazil; 3 Pediatrics, Universidade Federal do Rio de Janeiro, Rio de Janeiro, Brazil

## Introduction

The most widely used classification criteria for systemic lupus erythematous (SLE) are those derived and validated in adult patients, by the American College of Rheumatology (ACR). However, some of their limitations raise concerns, such as: inclusion of the most typical features only, possible duplication of highly correlated cutaneous manifestations, omission of many neurologic manifestations, inadequate quantification of urine protein by dipstick, lack of standardization for autoantibodies detection and, classification as SLE for patients that do not satisfy the immunologic disorder criterion. Alternative methods for SLE classification have been developed, such as the SLICC (Systemic Lupus International Collaborating Clinics) criteria.

## Objectives

We aimed to compare the performance of the new SLICC criteria with the 1997 ACR criteria in our juvenile SLE patients.

## Methods

Cases were JSLE and controls were patients with other rheumatic diseases, attending a tertiary center in the past 10 years. Data were retrospectively collected, to establish the ACR and SLICC criteria fulfilled, at first visit and within one year of follow-up. A consensus diagnosis of JSLE, established by the same group of highly experienced pediatric rheumatologists was chosen as the standard of reference. Statistical analyses were performed with SPSS 20.0 for *Windows.* Difference was regarded as statistically significant when p < 0.05.

## Results

Both sets of classification criteria were analyzed in 81 JSLE patients and 92 controls. There was a sharp increase in sensitivity and prevalence of all criteria within one year of follow-up. The application of SLICC criteria resulted in higher sensitivity (82.7% versus 58%, p < 0.001) at first visit, but similar specificity at both follow-up times (p = 1). There was no statistically significant difference regarding the number of misdiagnosis, both at first visit and within one year of follow up. Accuracy was higher for SLICC criteria at both periods.

## Conclusion

The main strength of this study was the comparison between the ACR and the new SLICC criteria, in a JSLE population at different moments of follow-up. In this JSLE population, the SLICC criteria performed best in terms of sensitivity at both observation periods. Despite this, it is still only 82.7% SLICC sensitivity at first visit, which means that, on average, one in five cases of JSLE would be missed. Considering the peculiarities of JSLE (higher frequency of atypical presentations and more severe presentation and evolution) and taking into account that these two criteria sets were developed and validated in adults, it should be considered modifications to increase early diagnostic sensitivity in JSLE.

## Disclosure of interest

None declared

